# *Imagin*: An Integrase-Like Gene Conserved Across Malacostracan Crustaceans Derived From a Ginger1 DNA Transposon

**DOI:** 10.1093/gbe/evag010

**Published:** 2026-01-16

**Authors:** Liyuan Hao, Satoshi Kawato, Reiko Nozaki, Miho Furukawa, Hidehiro Kondo, Ikuo Hirono

**Affiliations:** Laboratory of Genome Science, Tokyo University of Marine Science and Technology, Tokyo, Japan; Laboratory of Genome Science, Tokyo University of Marine Science and Technology, Tokyo, Japan; Laboratory of Genome Science, Tokyo University of Marine Science and Technology, Tokyo, Japan; Laboratory of Genome Science, Tokyo University of Marine Science and Technology, Tokyo, Japan; Laboratory of Genome Science, Tokyo University of Marine Science and Technology, Tokyo, Japan; Laboratory of Genome Science, Tokyo University of Marine Science and Technology, Tokyo, Japan

**Keywords:** Decapoda, integrase, kuruma shrimp, transposable elements, molecular domestication, ginger1

## Abstract

Domestication of transposable elements has been extensively documented in vertebrates, but few examples have been reported in nonmodel organisms, particularly crustaceans. Here, we present *Imagin* (*I*ntegrase-like gene in *MA*lacostracans derived from *GIN*ger1), a gene family derived from a *Ginger1* DNA transposon domesticated in the common ancestor of malacostracan crustaceans over 400 million years ago. We discovered *Imagin* in the kuruma shrimp *Penaeus japonicus* as a single-copy, multiexon gene residing within a conserved intron of the *methylmalonyl-CoA mutase* (*MMUT*) gene. Comprehensive phylogenetic and structural analyses demonstrate that while *Imagin* orthologs are under strong purifying selection and retain the conserved H2C2 zinc-finger domain and integrase core, they have ubiquitously lost the catalytic DDE triad essential for endonuclease activity. These structural features indicate that *Imagin* has undergone molecular exaptation, abandoning its ancestral mobility for a host function. Consistent with this loss of enzymatic capacity, PjImagin protein accumulates predominantly in the cytosol of oocytes during early development, rather than the nucleus. This localization pattern implies that the gene has been co-opted for a noncatalytic role, potentially involving nucleic acid binding, during female gonadal development in penaeid shrimp. Furthermore, transcriptome data revealed divergent expression profiles across lineages, where *Imagin* is enriched in the ovaries of penaeid shrimp but predominantly in the testes of other decapods, such as crabs and lobsters. *Imagin* thus represents a novel case of TE evolution, illustrating a complex history of ancient domestication followed by structural remodeling and regulatory subfunctionalization.

Significance statement Molecular domestication turns genomic parasites into functional host genes, yet clear examples of their subsequent evolutionary divergence remain rare. We identified *Imagin*, an integrase-like gene domesticated from a *Ginger1* transposon in the common ancestor of malacostracan crustaceans over 400 million years ago. Our findings suggest that this gene underwent molecular exaptation for a role in reproduction. Although the protein lost its original endonuclease activity, it is widely expressed in gonadal tissues. *Imagin* further exhibits regulatory subfunctionalization where its expression evolved divergently across taxa. The gene is enriched in the ovaries of penaeid shrimp, whereas in other decapods such as crabs and lobsters, expression is higher in the testes. This study highlights the complex evolution of a domesticated transposable element from initial capture and exaptation to functional diversification across diverse host species.

## Introduction

Transposable elements (TEs) are genetic elements that move within host genomes (Bourque et al. 2018). Although TEs are often regarded as “parasites” threatening genome integrity, they sometimes provide raw materials for molecular novelties in the host (Brandt et al. 2005). TE-originated host genes represent examples of molecular exaptation, or co-option, whereby proteins acquire biological roles distinct from their original functions following domestication by a new host.

TE domestication is especially well documented in vertebrates. One of the best-known examples is syncytin, a cell–cell fusion protein that plays a crucial role in the development of placenta in humans and closely related primates (Blond et al. 1999; Mi et al. 2000). Syncytin is derived from the *env* gene of the human endogenous retrovirus *HERV-W*. Another retrotransposon-derived gene, *Peg10*, is more widely distributed among mammals and is also essential for placental development (Ono et al. 2006, 2001). RAG1 and RAG2 recombinases, essential for V(D)J recombination, are derived from a Transib DNA transposon rather than a retrotransposon. They generate immune diversity by rearranging gene segments in immunoglobulin and T-cell receptor genes (Gellert 2002; Kapitonov and Jurka 2005; Zhang et al. 2019b). Some domesticated elements, like Lyosin (Kitao et al. 2025) and NYNRIN (NYN domain and Retroviral Integrase Containing; also known as CGIN1), are fused to cellular genes.

During the annotation process of a draft genome assembly of the kuruma shrimp *Penaeus japonicus* (Kawato et al. 2021), we discovered a multiexon gene encoding an integrase-like protein, which intrigued us because it possesses introns despite its apparently TE origin. Preliminary searches against publicly available decapod genomes and transcriptomes indicated that this gene is highly conserved among decapods, suggesting ancient origins and potential functional importance. The combination of integrase-like domains and stable conservation indicated possible molecular domestication, motivating us to investigate its evolutionary history and functional role.

## Results

### Discovery and Structure of an Integrase-like Gene (*PjImagin*)

**Fig. 1.**
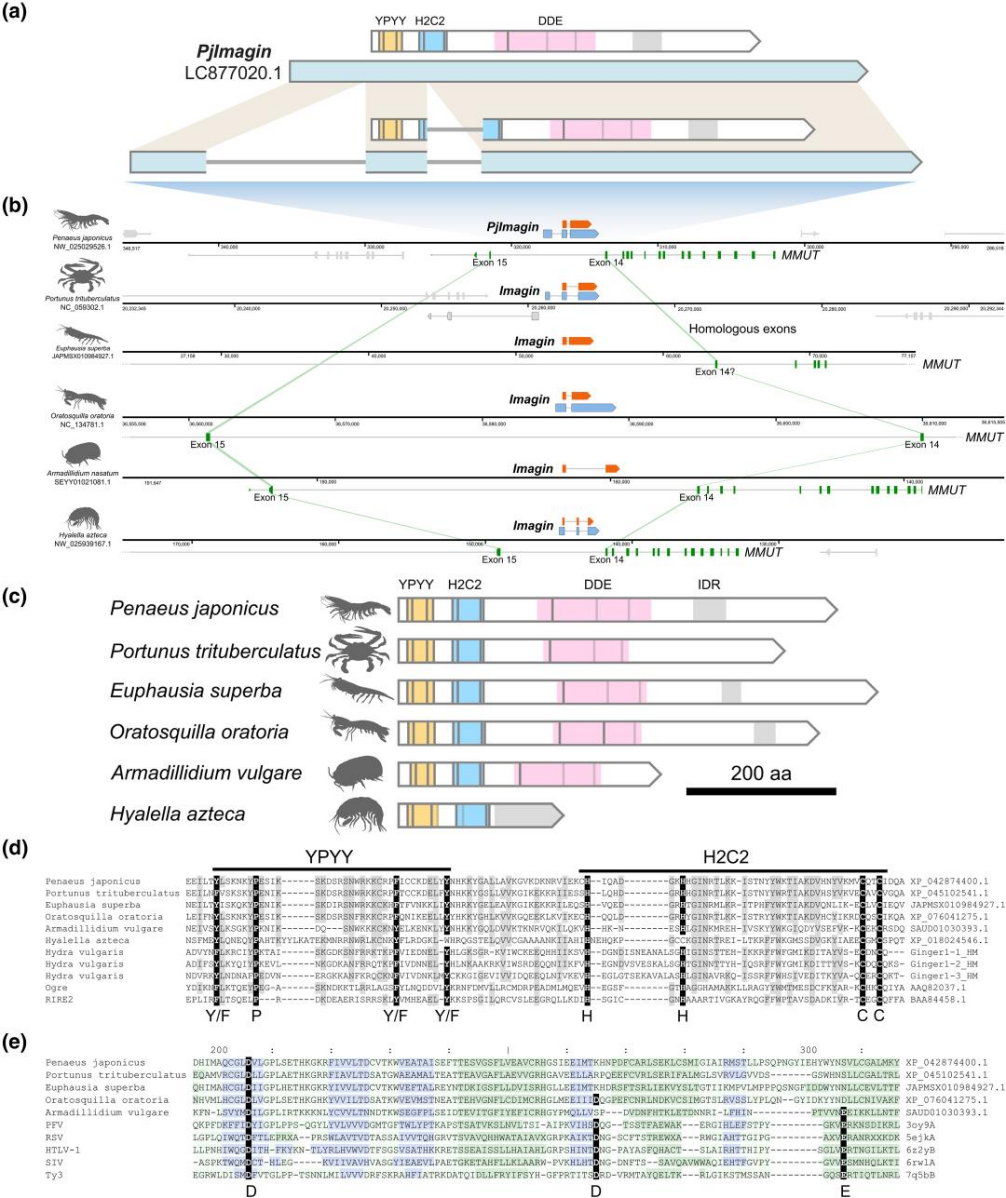
*Imagin*, a multiexon integrase-like gene in malacostracan crustaceans. a) Structure of the *PjImagin* gene. The transcript is shown in light blue and the CDS in white. The YPYY motif is colored yellow, the H2C2 zinc finger is colored sky blue, the defunct DDE catalytic core is colored in pink, and the disordered region is shaded in gray. Gray vertical lines denote the positions of key residues denoted in panels c and d, with the last 2 lines being shaded to indicate the corresponding positions for the lost D and E residues (panel e). b) The genomic context of *PjImagin* and orthologs. *PjImagin* is flanked by exons 14 and 15 of *methylmalonyl-CoA mutase*, *mitochondrial-like* (*MMUT*) gene, which runs on the opposite strand. Note that the *Imagin* gene of the swimming crab (*Portunus trituberculatus*) lies on an unrelated position. *MMUT* CDS is colored green, and *MMUT* exons corresponding to exons 14 and 15 of *P. japonicus MMUT* are connected with light green ribbons. CDS of other genes are shaded gray. c) Domain architectures of malacostracan Imagin proteins. The coloring scheme follows that of panel (a). d) Conservation of the YPYY motif and the H2C2 (HHCC) zinc-finger domain in integrase proteins. Species names are listed on the left, with protein accession numbers provided on the right. Entries Ginger1-1_HM, Ginger1-2_HM, and Ginger1-3_HM were adopted from Bao et al. (2010). The host species for these 3 entries was described as *Hydra magnipapillata* in Bao et al. (2010), but we opted for the species name *H. vulgaris*, deferring to NCBI Taxonomy (NCBI:txid6087). e) Loss of the DDE catalytic residues in crustacean Imagin proteins. Species names are listed on the left, with protein accession numbers provided on the right. Note that some residues may be missing from this alignment because regions containing long gaps were automatically suppressed by DALI. Each residue is shaded by background color according to the secondary structure assignments by DSSP: light green: loop; dark green: helix; blue: strand. Conserved aspartic acid (d) and glutamic acid (e) residues comprising the DDE motif are highlighted in black. PFV, prototype foamy virus; RSV, Rous sarcoma virus; HTLV-1, human T-lymphotropic virus 1; SIV, simian immunodeficiency virus; Ty3, Ty3 retrotransposon.

During the genome annotation of the kuruma shrimp *P. japonicus*, we identified a multiexon gene encoding a protein exhibiting similarity to transposases. We named this gene *PjImagin* (*P. japonicus Imagin*; LOC122254663). The full-length transcript of *PjImagin* (DDBJ Accession no. LC877020.1) was 2,623 nucleotides (nt) in length, encoding a 585-amino acid (aa) predicted protein with an estimated molecular mass of 64.9 kDa and an isoelectric point (pI) of 7.60. The gene comprises 3 exons, with the first exon consisting entirely of the 5′-untranslated region (UTR), and lies between exons 14 and 15 of *methylmalonyl-CoA mutase*, *mitochondrial-like* gene (*MMUT*; LOC122254656) (Fig. 1b; Table S1).

A domain search using HMMER3 revealed a H2C2 zinc-finger domain (PF17921) and a transposase-like catalytic core (IPR001584), both of which are characteristic of DDE transposases and integrases seen in retroelements and certain DNA transposons (Esposito and Craigie 1999) (Fig. 1a). However, structural alignment with representative integrases indicates that *PjImagin* and its orthologs in other crustaceans lack the conserved aspartic acid (D) and glutamic acid (E) residues that constitute the canonical DDE catalytic triad (Fig. 1e; Supplementary Data S1) (Nesmelova and Hackett 2010). This suggests that the Imagin protein has lost its enzymatic activity, at least with respect to the canonical DDE-dependent mechanism. Upstream of the zinc-finger domain lies the “YPYY motif”, which is found in a subset of retroelements and DNA transposons (Bao et al. 2010).

*PjImagin* orthologs and their genomic contexts are highly conserved across decapod crustaceans (Table 1; Fig. S1; Supplementary Data S2). The d*N*/d*S* ratios of *Imagin* orthologs in decapods ranged from 0.05 to 0.13, indicating that *Imagin* orthologs are under purifying selection (Table 2; Supplementary Data S3; Table S10). Conservation of the *Imagin* coding region and UTRs, particularly in *Penaeus* spp., suggests that these noncoding elements have critical regulatory roles (Good 1995). The extensive conservation of regulatory regions may reflect species-specific requirements for gene expression control, such as sex-biased expression patterns (Ellegren and Parsch 2007).

**Table 1:** Imagin sequences identified from malacostracan crustaceans

Order	Suborder	Infraorder	Family	Species	Genome	Transcript	Protein	Reference
Decapoda	Dendrobranchiata	…	Penaeidae	*Penaeus japonicus*	NW_025029526.1	XM_043018466.1, LC877020.1	XP_042874400.1	Kawato et al. (2021)
…	…	…	…	*Penaeus vannamei*	NW_020872734.1	XM_027351321.1	XP_027207122.1	Zhang et al. (2019a)
…	…	…	…	*Penaeus monodon*	NC_051387.1	XM_037929098.1	XP_037785026.1	Uengwetwanit et al. (2021)
…	…	…	…	*Penaeus chinensis*	NC_061830.1	XM_047621599.1	XP_047477555.1	Yuan et al. (2021)
…	…	…	…	*Penaeus indicus*	NW_026949151.1	XM_063750415.1	XP_063606485.1	Katneni et al. (2022)
…	…	…	…	*Penaeus latisulcatus*	–	GGTT01000830.1	–	Huerlimann et al. (2020)
…	…	…	…	*Penaeus longistylus*	–	GGTU01001009.1	–	Huerlimann et al. (2020)
…	…	…	…	*Penaeus semisulcatus*	–	GGTV01001257.1	–	Huerlimann et al. (2020)
…	…	…	…	*Penaeus aztecus*	–	GEUA01008623.1	–	Johnson et al. (2016)
…	Pleocyemata	Caridea	Palaemonidae	*Palaemon carinicauda*	NC_090739.1	XM_068388552.1	XP_068244653.1	Wang et al. (2024a)
…	…	…	…	*Macrobrachium nipponense*	NC_087202.1	XM_064259868.1	XP_064115938.1	Jin et al. (2021)
…	…	…	…	*Macrobrachium rosenbergii*	NC_089752.1	–	–	Zheng et al. (2024)
…	…	…	…	*Macrobrachium novaehollandiae*	–	GHDW01049199.1	…	Rahi et al. (2019)
…	…	…	…	*Macrobrachium tolmerum*	–	GHDQ01045443.1	–	Rahi et al. (2019)
…	…	…	Pandalidae	*Pandalus borealis*	JAVIII010019169.1	–	–	Bourret et al. (2024)
…	…	…	Crangonidae	*Crangon crangon*	–	GKAF01001613.1	–	Raghavan et al. (2023)
…	…	…	Atyidae	*Halocaridina rubra*	JAXCGZ010009481.1	–	–	PRJNA1037494
…	…	…	…	*Halocaridinides trigonophthalma*	–	GHBI01031222.1	–	Genomic Resources Development Consortium et al. (2014)
…	…	…	…	*Neocaridina denticulata*	BAABUL010001016.1	–	–	Adachi et al. (2025)
…	…	…	…	*Caridina multidentata*	BDMR012730770.1	–	–	Sasaki et al. (2017)
…	…	Achelata	Palinuridae	*Panulirus ornatus*	NC_092244.1	XM_071675491.1	XP_071531592.1	Ren et al. (2024)
…	…	…	…	*Panulirus gracilis*	JAUUEG010005451.1	–	–	Baeza et al. (2024)
…	…	…	…	*Panulirus versicolor*	JAUUDZ010013417.1	–	–	Baeza et al. (2024)
…	…	…	…	*Panulirus inflatus*	JAUUDY010602921.1	–	–	Baeza et al. (2024)
…	…	…	…	*Panulirus laevicauda*	JAUTXX010152419.1	–	–	Baeza et al. (2024)
…	…	…	…	*Panulirus homarus*	JAUUEF010021749.1	–	–	Baeza et al. (2024)
…	…	…	…	*Panulirus argus*	–	GHUJ01013554.1	–	Ernst et al. (2020)
…	…	…	…	*Jasus edwardsii*	–	GGHM01141313.1, GGHM01179921.1	–	Souza et al. (2018)
…	…	Astacidea	Homaridae	*Homarus americanus*	NW_024725521.1	XM_042364571.1	XP_042220505.1	Polinski et al. (2021)
…	…	…	…	*Homarus gammarus*	CAUDMG010003042.1	–	…	Paris et al. (2025)
…	…	…	Parastacidae	*Cherax quadricarinatus*	NC_091364.1	XM_070100566.1	XP_069956667.1	Liu et al. (2024)
…	…	…	…	*Cherax destructor*	WNWK01008214.1	–	–	Austin et al. (2022)
…	…	…	Cambaridae	*Procambarus clarkii*	NC_059639.1	XM_045765530.1	XP_045621486.1	Xu et al. (2021)
…	…	…	…	*Procambarus virginalis*	–	GJEC01212686.1	–	Boštjančić et al. (2022)
…	…	…	…	*Procambarus erythrops*	–	GIVA01013313.1	–	Carneiro et al. (2021)
…	…	Anomura	Porcellanidae	*Petrolisthes cinctipes*	JAWQEG010005873.1	–	KAK3856478.1	Angst et al. (2024)
…	…	…	…	*Petrolisthes manimaculis*	JAWZYT010003468.1	–	KAK4298242.1	Angst et al. (2024)
…	…	…	Coenobitidae	*Coenobita brevimanus*	JAVRFU020000059.1	–	–	Wang et al. (2024c)
…	…	…	Lithodinae	*Paralithodes platypus*	JAVRFV010000049.1	–	–	Tang et al. (2021)
…	…	…	…	*Paralithodes camtschaticus*	–	GHJC01074738.1	–	Stillman et al. (2020)
…	…	Brachyura	Varunidae	*Eriocheir sinensis*	NC_066510.1	XM_050860434.1	XP_050716391.1	Wang et al. (2022)
…	…	…	…	*Hemigrapsus takanoi*	GKPI01496440.1	–	–	PRJNA1018655
…	…	…	Oregoniidae	*Chionoecetes opilio*	JACEEZ010023931.1	–	–	PRJNA602365
…	…	…	Cancridae	*Cancer borealis*	JBCDWE010000017.1	–	–	Polinski et al. (2025)
…	…	…	…	*Metacarcinus magister*	JAPQNM010000040.1	–	–	PRJNA902360
…	…	…	Carcinidae	*Carcinus maenas*	–	GFYV01193636.1	–	Oliphant et al. (2018)
…	…	…	Portunidae	*Callinectes sapidus*	JAOPJN010000262.1	–	–	PRJNA773940
…	…	…	…	*Scylla paramamosain*	NC_087162.1	XM_064014698.1	XP_063870768.1	Zhang et al. (2024)
…	…	…	…	*Portunus trituberculatus*	NC_059302.1	XM_045246606.1	XP_045102541.1	Tang et al. (2020)
…	…	…	…	*Thalamita danae*	–	GKPJ01278730.1	–	PRJNA1018655
Euphausiacea	…	…	Euphausiidae	*Euphausia superba*	JAPMSX010984927.1	–	–	PRJNA867116
…	…	…	…	*Meganyctiphanes norvegica*	CAXKWB010041792.1	–	–	Unneberg et al. (2024)
Stomatopoda	…	…	Squillidae	*Oratosquilla oratoria*	NC_134781.1	XM_076185160.1	XP_076041275.1	Zhang et al. (2025a)
Isopoda	Oniscidea	…	Armadillidiidae	*Armadillidium vulgare*	SAUD01030393.1	–	–	Chebbi et al. (2019)
…	…	…	…	*Armadillidium nasatum*	SEYY01021081.1	–	–	Becking et al. (2019)
…	…	…	Trichoniscidae	*Trichoniscus matulici*	–	GKUF01008836.1	–	Jovović et al. (2024)
…	…	…	…	*Hyloniscus beckeri*	–	GKTX01069465.1	–	Jovović et al. (2024)
…	Cymothoida	…	Cirolanidae	*Bathynomus jamesi*	JAJOZX010000594.1	–	–	Yuan et al. (2022)
Amphipoda	Talitrida	…	Hyalellidae	*Hyalella azteca*	NW_025939167.1	XM_018169057.2	XP_018024546.1	Poynton et al. (2018)
…	…	…	Hyalidae	*Parhyale hawaiensis*	–	GFVL01019626.1	–	Hunt et al. (2019)
…	Talitroidea	…	Talitridae	*Talitrus saltator*	–	GDUJ01036696.1	–	O’Grady et al. (2016)
…	…	…	…	*Trinorchestia longiramus*	VCRD01006928.1	–	KAF2348077.1	Patra et al. (2020)
…	…	…	…	*Morinoia aosen*	JASJYW010000022.1	–	–	Liu et al. (2023)
…	Gammaridea	…	Hirondelleidae	*Hirondellea gigas*	–	GEZX01077614.1	–	Lan et al. (2017)

**Table 2:** d*N*/d*S* values for Imagin CDSs in decapod crustacean**s**

Taxon	Sequences	Codons	Total Tree length (subs/site)	Log(L)	AIC-c	Estimated parameters	d*N*/d*S* (95% profile CI)
*Penaeus*	9	586	0.185	−3880.06	7820.48	30	0.0574 (0.0420 to 0.0761)
*Macrobrachium*	4	587	0.08	−3054.74	6149.84	20	0.1332 (0.0960 to 0.1789)
Astacidea	5	611	0.194	−3615.76	7275.85	22	0.1245 (0.1006 to 0.1520)
*Panulirus*	7	611	0.057	−2975.32	6000.94	25	0.0603 (0.0354 to 0.0949)
Portunidae	4	524	0.174	−3252.48	6545.37	20	0.1255 (0.0972 to 0.1585)

See Table S10 for the details of the sequences used in each analysis. The CDS alignments are available as Supplementary Data S3.

**Fig. 2.**
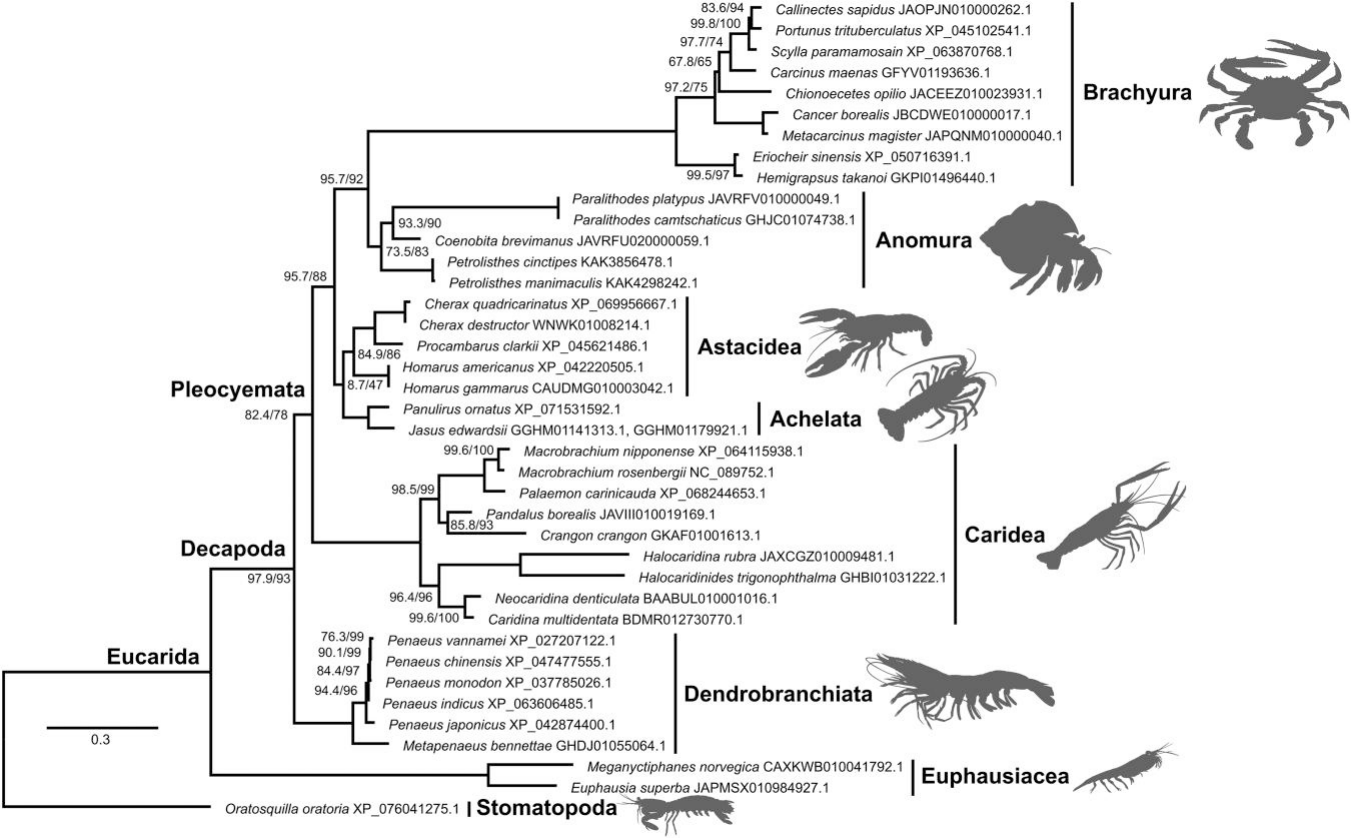
Phylogenetic analysis of decapod imagin proteins. A total of 463 sites were used for the maximum-likelihood phylogenetic analysis using IQ-TREE v3.0.1 (substitution model: Q.MAMMAL + F + I + G4). The slashed values beside the nodes indicate the support values for the ultrafast bootstrap test, followed by the SH-like approximate likelihood ratio test (1,000 trials each). The bar indicates amino acid substitutions per site. Euphausiacea and Stomatopoda were used as the outgroup. Imagin proteins from brachyuran crabs formed a long branch relative to the other taxa, suggesting accelerated evolution following the translocation event in this lineage.

Brachyuran *Imagin* orthologs are absent from the canonical location and reside in a different genomic region (Fig. 1b, Fig. S1), but their orthology is supported by the facts that: (i) they are single-copy genes (Table S2), and (ii) the phylogenetic tree drawn using the *Imagin* orthologs conforms to the species phylogeny of decapods (Fig. 2; Supplementary Data S4) (Wolfe et al. 2019), although brachyuran crabs formed an exceptionally long branch relative to the other taxa. This could be due to accelerated evolution of the *Imagin* gene in brachyuran crabs, as suggested by the divergent genomic location of the *Imagin* gene in these species.

The remarkable conservation in decapods prompted us to explore *Imagin* orthologs across malacostracan crustaceans. TBLASTN searches querying the PjImagin protein sequence against publicly available malacostracan genomes readily identified Imagin orthologs from krills (*Euphausia superba* and *Meganyctiphanes norvegica*; order Euphausiacea) (Unneberg et al. 2024) and the mantis crab (*Oratosquilla oratoria*; order Stomatopoda) (Table 1; Table S3) (Zhang et al. 2025a), with all lying between exons 14 and 15 of the *MMUT* gene (Fig. 1b; Fig. S1; Table S1). Krill and mantis crab Imagin proteins closely resemble decapod orthologs in terms of domain architecture and lengths.

Imagin orthologs were present in isopods and amphipods (peracarids) between exons 14 and 15 of the *MMUT* gene, but *Imagin* orthologs in these organisms were truncated relative to those of decapods (Fig. 1b and c; Fig. S1; Table S4 to S8; Supplementary Data S2 and S5). In amphipods, the YPYY motif and the H2C2 zinc finger were degenerated, and the DDE integrase domain has been completely lost, with the C-terminal substituted by intrinsically disordered region.

We could not examine the presence of *Imagin* orthologs in other malacostracan lineages, such as Leptostraca, Mysida, and Tanaidacea, but this was due to the lack of high-quality genome assemblies and does not exclude the possibility that *Imagin* orthologs exist in these lineages.

Collectively, these findings demonstrate that *Imagin* domestication in an intronic region between exons 14 and 15 of *MMUT* gene took place at least before the divergence of major malacostracan clades, which likely dates back to the Cambrian to Ordovician (490 to 440 million years ago) (Bernot et al. 2023).

### *Imagin* Originated From a Ginger1 DNA Transposon: Naming and Etymology

**Fig. 3.**
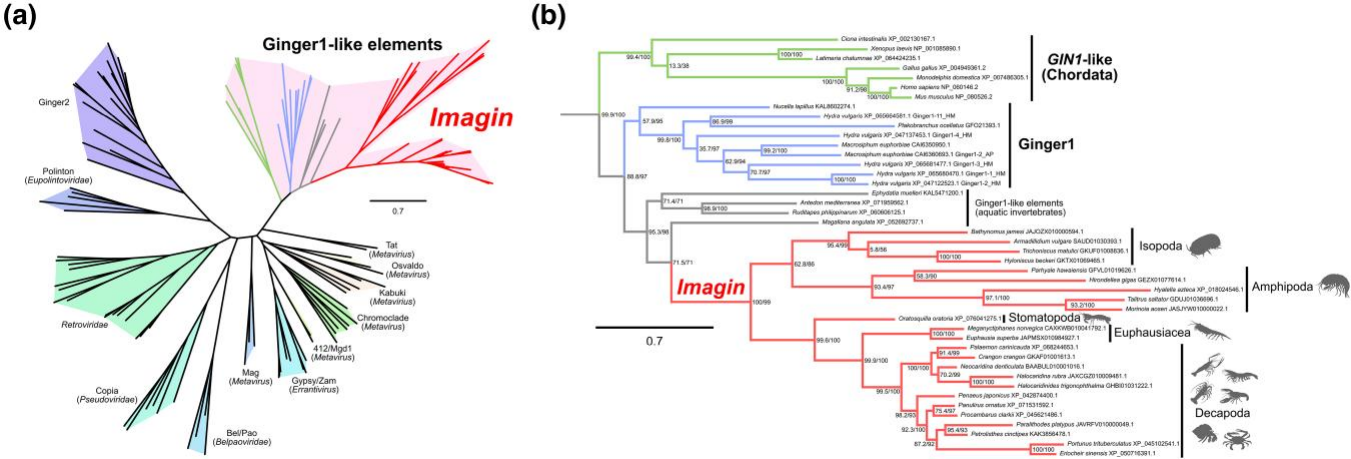
*Imagin* is a domesticated Ginger1 DNA transposon. a) Maximum-likelihood phylogenetic tree of 121 DDE integrases and derivatives (136 sites; model: VT + R6). b) Subtree of a) showing the phylogenetic relationships of Ginger1-like elements. The bar beside the tree indicates amino acid substitutions per site. The slashed values beside the nodes indicate the support values for the ultrafast bootstrap test, followed by the SH-like approximate likelihood ratio test (1,000 trials each).

To investigate the phylogenetic origins of *Imagin*, we built a maximum-likelihood phylogenetic tree of DDE integrases from LTR retrotransposons and retroviruses (Kojima 2019). The tree resolved major clades of retroviruses and transposons and placed Imagin orthologs within the branch made up of Ginger1 (“*Gypsy* INteGrasE Related 1”)-like elements, a family of multiexon DNA transposons phylogenetically related to retroelement integrases (Bao et al. 2010; Marín 2010) (Fig. 3; Supplementary Data S6; Table S9). This tree placed human *GIN1* (Lloréns and Marín 2001) in a clade distinct from Imagin, confirming their independent evolutionary histories. The conservation of YPYY motif among Imagin orthologs (Fig. 1d) also aligns well with phylogenetic relationships.

Based on all these findings, we formally named this gene family “*Imagin*” (***I***ntegrase in ***ma***lacostracans originating from ***Gin***ger1). The omission of the final “e” from “imagine” alludes to the structural loss of the conserved glutamate (E) residue in the DDE catalytic triad described above. In hindsight, *Imagin* possessing introns was not surprising given that this gene originated from a multiexon DNA transposon. The Imagin genes have defunct DDE motifs and lack the inverted repeats, both of which are essential for mobility. The loss of these features was likely essential for the domestication event.

### PjImagin Protein Accumulates in the Cytosol of Developing Oocytes

**Fig. 4.**
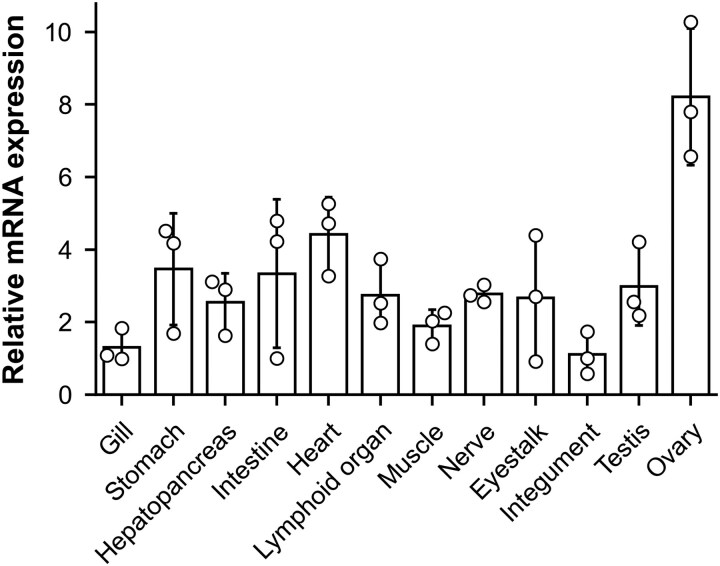
Expression of *PjImagin* in different tissues. The relative expression levels of *PjImagin* in different tissues of kuruma shrimp (*n* = 3). Expression values are visualized using barplots overlaid with beeswarm plots to show both distribution and individual data points.

We used *P. japonicus* as a model to analyze the expression and functions of *Imagin*. qPCR analysis revealed *PjImagin* expression was markedly elevated in the ovary, suggesting a role in female reproductive development (Fig. 4).

**Fig. 5.**
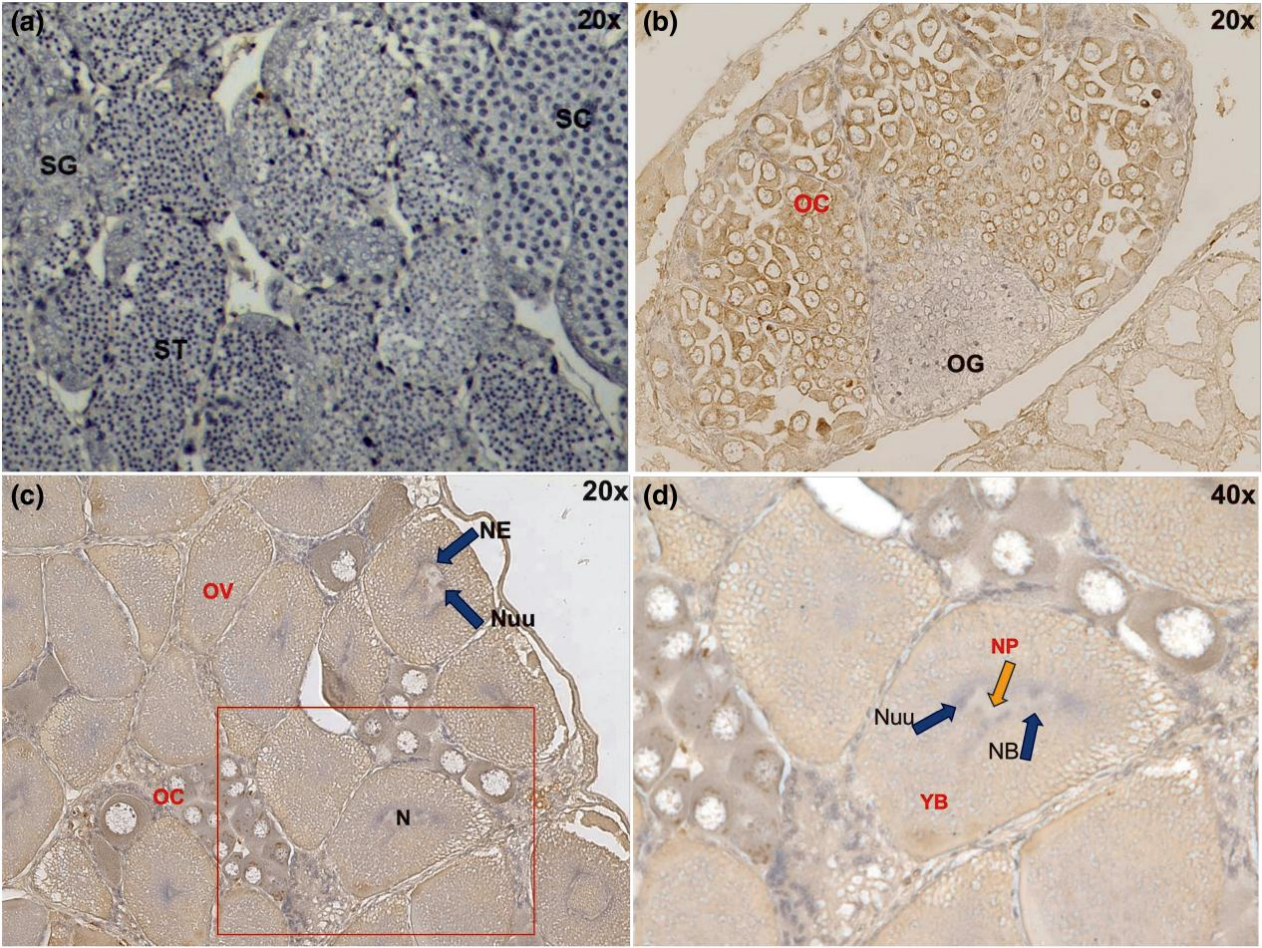
PjImagin accumulates in developing oocytes. Immunohistochemical staining was performed on gonadal sections to examine the distribution of PjImagin. a) A testis section from a male shrimp showed no detectable DAB-positive signal, while ovarian sections from female shrimps showed clear localization of PjImagin. b) The ovary from a 10 g female shrimp was observed under 20× magnification, and c and d) the ovary from a 25 g female shrimp was observed under both 20× and 40× magnifications. Brown DAB staining indicates PjImagin localization. Cell stages with positive signals are labeled in red, while negative ones are labeled in black. All sections were counterstained with hematoxylin. Abbreviations: OG, oogonium; OC, oocyte; OV, mature ovum; YB, yolk body; N, nucleus; NE, nuclear envelope; Nuu, nucleolus; NP, nucleoplasm; SG, spermatogonium; SC, spermatocyte; ST, spermatid.

We next performed immunohistochemistry to examine the cellular localization of PjImagin protein in kuruma shrimp gonads. No signals were detected from the testis, suggesting that PjImagin protein is not expressed in the male gonad (Fig. 5a). In the immature ovary (BW: 10 g; Fig. 5b), brown positive signals were detected exclusively in the oocytes while oogonia were negative, suggesting that PjImagin accumulation takes place in female germline cells undergoing meiosis. Notably, positive signals were present in the cytosol and not in the nucleus of oocytes. PjImagin accumulation is further pronounced in more developed oocytes and mature ova (BW: 25 g; Fig. 5c and d). In mature ova, positive signals were detected in yolk and nucleoplasm, while the nucleoli or nuclear membrane remained negative (Fig. 5d).

### Contrasting Expression of *Imagin* Orthologs Between Two Decapod Suborders

**Fig. 6.**
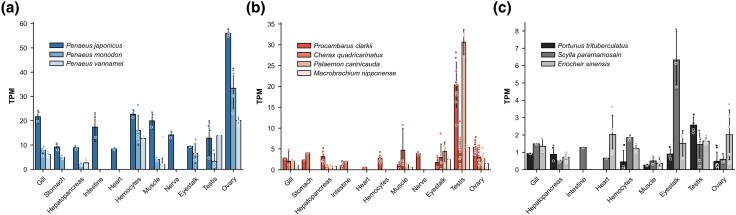
TPM expression levels of *Imagin* across decapod species and tissues. Expression values are visualized using barplots overlaid with beeswarm plots to show both distribution and individual data points. Imagin expression is highest in the ovary of penaeid shrimp (a) but enriched in the testes of prawns and crayfish (b) as well as crabs (c), indicating tissue-specific roles across species. See Table S11 for the details of the datasets used.

To explore the tissue distribution and functions of *Imagin* orthologs in other decapods, we turned to public RNA-seq data and reference genome assemblies (Table S11). In penaeid shrimps, *Imagin* expression was detected predominantly in the ovary (Fig. 6a), aligning with our qPCR data in *P. japonicus.* In contrast, in other decapod crustaceans, such as crabs and lobsters, *Imagin* showed high expression in the testis rather than ovary (Fig. 6b and c). The marked difference in tissue specificity between penaeid shrimp and other decapods implies a functional divergence of *Imagin* in reproductive processes across crustacean lineages. Collectively, these observations suggest that, although *Imagin* orthologs are essential for reproductive biology in decapods, their precise roles may vary among species.

## Discussion

TEs are ubiquitous in cellular genomes, and crustaceans are no exception. In fact, crustacean genomes are notoriously rich in repetitive sequences, which poses challenges for bioinformaticians working on these organisms. Despite the abundance of TEs, their biological significance, including the contribution of domesticated elements, has been poorly understood in crustaceans. From a reverse genetics perspective, this could be due to the difficulty in distinguishing domesticated elements from “parasitic” or “junk” elements in genome sequences. This challenge is further compounded by the difficulty of applying forward genetics approaches to these nonmodel organisms.

The discovery of *Imagin* was a serendipitous byproduct of a previous shrimp genome sequencing project (Kawato et al. 2021). The bioinformatic analyses were driven by the availability of high-quality genome and transcriptome data, which allowed us to identify orthologs, including their conserved genomic context. Bioinformatics-driven discovery of domesticated TEs requires careful examination of conservation across taxa as well as avoiding prematurely masking domesticated genes as repetitive elements.

*Imagin* originates from an integrase, which is a family of DNA recombination enzymes responsible for the insertion of genetic elements into host genomes (Collis and Hall 1992; Masuda 2011). Integrases are characterized by conserved functional domains, such as the H2C2 zinc finger and the catalytic DDE motif (Nowotny 2009). The domestication of integrase by the host can give rise to novel biological functions (Miller et al. 1997; Chalopin et al. 2012; Koonin and Krupovic 2018). Importantly, when TEs are domesticated by the new host, crucial factors are the biochemical properties of the protein stemming from the structure, rather than the biological role(s) it played in the original context.

While *Imagin* orthologs are widely conserved across malacostracan crustaceans, they lack key DDE residues required for enzymatic activity, which suggests that it does not function as a canonical integrase. Regardless, the overall structural integrity of the integrase core domain, or in general, the RNase H-like fold (Majorek et al. 2014), is well preserved. This raises the possibility that Imagin acts as a nucleic acid-binding protein or a scaffolding protein mediating protein–protein interactions, likely cooperating with the N-terminal H2C2 zinc-finger domain that aids multimerization (Zheng et al. 1996; Lee et al. 1997), rather than functioning as an enzyme.

The accumulation of PjImagin in the cytoplasm of developing oocytes is consistent with this hypothesis. Cytoplasmic proteins in crustacean oocytes often participate in maternal mRNA regulation (Howley and Ho 2000), translational control (Richter and Lasko 2011; Sengseng et al. 2023), or yolk processing (Kessel 1968), all of which are essential for proper embryonic development. PjImagin's association with yolk bodies suggests a role in vitellogenesis (Tsukimura 2001) or in modulating genes related to gonadal maturation (Sellars et al. 2015; Potiyanadech et al. 2023). PjImagin may contribute to some of these functions; for instance, functioning as a carrier or storage platform for maternal RNAs, where high protein abundance is required to sequester or regulate nucleic acid targets. Examples of domesticated TEs serving as an RNA-binding protein include *Jerky* in mammals (Toth et al. 1995; Liu et al. 2002, 2003), although the RNA-binding domain of Jerky is a tandem repeat of homeodomain-like helix-turn-helix, which is different from Imagin.

While the maternal RNA-binding protein hypothesis could explain the *Imagin* expression dynamics in penaeid shrimps (Suborder Dendrobranchiata), *Imagin* showed highest expression in the testis of other decapods (Suborder Pleocyemata). This is rather intriguing and warrants further investigation, as it suggests the subfunctionalization of *Imagin* across the 2 suborders. The accelerated evolution observed in brachyuran Imagin orthologs (Fig. 2) may be associated with their translocation from the conserved *MMUT* intron, which likely released the gene from the strict evolutionary constraints imposed by the host gene.

Some domesticated TEs are responsible for controlling other TEs in the host genome. Although we cannot rule out the possibility that *Imagin* somehow contributes to the maintenance of host genome integrity, it is unlikely that *Imagin* is directly interacting with other TEs. If *Imagin* were an effector gene actively engaged in an “evolutionary arms race” against parasitic elements, its evolutionary trend should be characterized by diversification and divergence, which would involve gene duplication events and positive selection. Regardless, *Imagin* has remained a single-copy gene and, at least in decapods, has been under purifying selection. Even in brachyuran crabs, where *Imagin* has translocated from the conserved intron that could have potentially imposed constraints on duplication events, the gene has remained single copy. Given these observations, if *Imagin* plays a role in TE control, it would be a regulatory one like that of *ALP1* in land plants, which has also been under purifying selection (Liang et al. 2015).

Despite deep conservation at the locus level, amphipod *Imagin* has undergone drastic sequence divergence compared to other lineages. Amphipod *Imagin* has completely lost the integrase core and terminates with a low-complexity repeat, which is predicted to be an intrinsically disordered region; even the YPYY and the H2C2 motifs on the N-terminal are degraded (Fig. 1). Although we can only speculate the functions of this divergent Imagin protein, its strict conservation as a protein-coding gene suggests this gene is essential in amphipods. Regardless of its biological roles, the structural plasticity of *Imagin* in different host lineages illustrates how a domesticated transposon can evolve to serve host-specific needs, once it has been liberated from ancestral functional constraints.

While *Imagin* is conserved across malacostracan crustaceans, it has undergone notable structural and regulatory divergence. While originally derived from a mobile element, *Imagin* appears to have been co-opted into reproductive processes in a lineage-specific manner. Further studies, including RNA interference experiments to investigate the functional role of *PjImagin*, as well as broader species comparisons, are needed to determine whether integrase-like elements contribute to crustacean reproduction and whether their domestication represents an evolutionary trend.

## Materials and Methods

### Experimental Animals

Kuruma shrimp (average body weight: 15 g) were obtained from a commercial shrimp farm in Okinawa, Japan. Shrimp were maintained in a tank with a water recirculating system at 25 °C and a salinity of 29 to 32 ppt. Shrimp were acclimated for 3 d prior to downstream experiments.

### Cloning of the Full-length *PjImagin* cDNA

Total RNA was extracted from the ovaries of kuruma shrimp using RNAiso Plus reagent (Takara Bio Inc., Japan). The full-length cDNA sequence was cloned by 5′- and 3′-rapid amplification of cDNA ends (RACE)-PCR using the SMARTer RACE 5′/3′ Kit (Clontech, Japan), following the manufacturer's protocol. The primers used in this study are found in Table 3.

**Table 3:** Primers used in this study

Primer	Sequence (5′—to 3′)
PjEF1a_F	ATGGTTGTCAACTTTGCCCC
PjEF1a_R	TTGACCTCCTTGATCACACC
PjEF1a_qPCR_F	ATTGCCACACCGCTCACA
PjEF1a_qPCR_R	TCGATCTTGGTCAGCAGTTCA
PjImagin_F	GCAGTGTGGACTAGATGTTC
PjImagin_R	CTCCTGCTCATCAGAGTAAG
PjImagin_qPCR_F	TCATCCACCCCAACCAACTC
PjImagin_qPCR_R	CAGATTGGAGGTTTGAGCCG
PjImagin_5RACE	ATTCCTCCTTCCGACTCTTCCTGAGATGTG
PjImagin_5RACE2	TATCCTTGACTCCCTTCACG
PjImagin_3RACE	CCAACTCTTCCCAGCATCAATCCAGTCAAG
PjImagin_3RACE2	CCATTCAATCCAACAGCTCG
PjImagin_3RACE3	ATCTGCTCTCAGTGATGATG
NdeI_PjImagin_antigen_F	catatgGGAACACAACATCCAGACAG
PjImagin_antigen_6His_R	tcaatgatgatgatgatgatgTGAGTCTCCGTACG

### Characterization of the *PjImagin* Transcript

A *PjImagin* transcript sequence (ICRK01003983.1) was identified from a *P. japonicus* transcriptome assembly (Kawato et al. 2021). The sequence was analyzed with SnapGene Viewer v. 5.3.2 (GSL Biotech, United States) and GENETYX version 11.0.4 (Software Development Co. Ltd, Japan). *PjImagin* orthologs in other decapod crustaceans were identified using NCBI BLAST (https://blast.ncbi.nlm.nih.gov/Blast.cgi).

Functional domains in the predicted protein sequences were predicted using HMMER (Eddy 2011) (https://www.ebi.ac.uk/Tools/hmmer/). The molecular size and theoretical isoelectric point of the protein were predicted using the ExPASy (Duvaud et al. 2021) Compute pI/Mw tool (https://web.expasy.org/compute_pi/).

### RNA Isolation and cDNA Synthesis

Gill, heart, epidermis, stomach, lymphoid organ, nerve, muscle, hepatopancreas, eye stalk, intestine, and gonads were collected from 6 apparently healthy shrimp. Total RNA was extracted using RNAiso Plus (Takara Bio Inc., Japan) following the manufacturer's instructions, precipitated with isopropanol, and resuspended in DEPC-treated distilled water. RNA concentration was measured by spectrophotometry (NanoDrop, Thermo Scientific, United States). cDNA synthesis was carried out using the High-Capacity cDNA Reverse Transcription Kit with RNase Inhibitor (Applied Biosystems, United States). The cDNA was stored at −20 °C until further use.

### Tissue Distribution Analysis by RT-qPCR

Before qPCR, the cDNA was further diluted to a final concentration of 10 ng/µL with nuclease-free distilled water (DW). Quantitative PCR was performed on StepOnePlus Real-Time PCR System (Applied Biosystems, United States). Each reaction was performed in a final volume of 20 μl containing 5 μl of cDNA sample as a template, 10 μl of THUNDERBIRD Next SYBR qPCR Mix (TOYOBO, Japan), 3.8 μl of nuclease-free water, and 0.6 μl each of sense primer and antisense primer (10 pM). The cycling program was initiated from 95 °C (5 min) for preincubation, followed by 40 cycles at 95 °C (5 s) and 60 °C (30 s). The relative expression of the *PjImagin* was calculated by the 2^−ΔΔCt^ comparative Ct method.

### Recombinant Protein Expression and Antibody Preparation

A partial coding sequence (CDS) of PjImagin (19.7 kDa; rPjImagin) was cloned into the pET-32a(+) expression vector and introduced into *E. coli* BL21(DE3) cells. rPjImagin expression was induced with IPTG, and the N-terminal 6×His-tagged recombinant protein was purified using Ni-NTA Agarose (QIAGEN, Germany) following the manufacturer's instructions.

The purified protein was used to generate a polyclonal antibody in rabbits. The specificity of the antibody was confirmed by Western blot analysis, in which the antibody specifically recognized the rPjImagin protein (data not shown).

### Immunohistochemistry

Kuruma shrimp (*P. japonicus*) with body weights of approximately 10 and 25 g were used. Shrimp were injected with Bouin's solution (Fujifilm, Japan) and then immersed in the same fixative at 4 °C for 24 h. Tissues were dehydrated through a graded ethanol series and embedded in paraffin (Bell and Lightner 1988). Longitudinal sections of the cephalothorax were cut at a thickness of 4 μm and mounted onto slides for immunohistochemical analysis.

The sections were rehydrated and treated with 3% hydrogen peroxide in methanol for 10 min at room temperature to block endogenous peroxidase activity. Antigen retrieval was carried out in a water bath at 90 °C for 20 min using HistoVT One (Ichida et al. 2021) (NACALAI Tesque Inc., Japan). After treatment, the slides were blocked with Block Ace (KAC Co., LTD, Japan) for 1 h at room temperature, then incubated with anti-rPjImagin rabbit polyclonal antibody (1:5,000 dilution in Can Get Signal Solution A; TOYOBO, Japan) at 4 °C overnight. The sections were washed 3 times for 5 min each with PBST.

Following incubation, the sections were treated with a ready-to-use Peroxidase Polymer Anti-Rabbit IgG reagent (VECTOR LABORATORIES, United States) for 30 min at room temperature. After the immunoreactions, the sections were washed 3 times with PBST (0.05% Tween-20 in PBS) for 5 min each. Signal development was performed using 3,3′-diaminobenzidine (DAB; VECTOR LABORATORIES, United States) as the chromogen, which produces a brown precipitate at sites of positive immunoreactivity. The sections were then counterstained with hematoxylin, resulting in blue coloration of nuclei and negative signals, mounted with Malinol (Muto Pure Chemicals Co., Ltd., viscosity: 750 cps), and observed under a BZ-X810 microscope (Keyence, Japan).

### Bioinformatic Analyses

See Supplementary Data S7 for the scripts used in this study.

### Expression Profiling Using Public RNA-seq Data

The reference genome assemblies of 9 decapod crustacean genomes (Table S11) were downloaded from the NCBI database (Zhang et al. 2019a; Tang et al. 2020; Jin et al. 2021; Kawato et al. 2021; Uengwetwanit et al. 2021; Xu et al. 2021; Liao et al. 2024; Liu et al. 2024; Zhang et al. 2024; Wang et al. 2024a). RNA sequencing (RNA-seq) reads from various organs (e.g. heart, liver, etc.) were retrieved from the NCBI Sequence Read Archive (SRA) (Jiang et al. 2014; Shen et al. 2014; Manfrin et al. 2015; Peng et al. 2015; Huerlimann et al. 2018; Tinwongger et al. 2019; Nong et al. 2020; Santos et al. 2020; Tang et al. 2020; Kawato et al. 2021; Jia et al. 2022; Li et al. 2022; Yang et al. 2022; Zhang et al. 2022a, 2022b; Hu et al. 2023; Jia et al. 2023; Ling et al. 2023; Smith et al. 2023; Sun et al. 2023; Wang et al. 2023; Chang et al. 2024; Jiang et al. 2024; Li et al. 2024; Liao et al. 2024; Wang et al. 2024b; Chen et al. 2025; Li et al. 2025; Wang et al. 2025; Xu et al. 2025; Zhang et al. 2025b) (Table S11). The raw RNA-seq data underwent preprocessing with fastp (Chen et al. 2018) to remove low-quality reads and adapter sequences. After the quality control, the cleaned reads were mapped to the reference genome using STAR (Dobin et al. 2013), and gene expression levels were quantified with RSEM (Li and Dewey 2011). Transcripts per million (TPM) values were calculated to assess gene expression across different tissues.

### Exploration of Imagin Orthologs in Malacostracan Genomes

Genomic scaffolds containing *Imagin* or *MMUT* genes were explored by querying crustacean Imagin and MMUT proteins against the NCBI WGS database (last accessed 2025 November). The query sequences varied depending on the strategy and the target. The selected scaffolds were downloaded, and malacostracan Imagin and MMUT proteins were aligned onto the scaffolds using miniprot v0.18-r281 (Li 2023). The resulting GFF3 files were parsed using gffread v0.12.7 (Pertea and Pertea 2020). Sequence integrity and completeness were analyzed visually using IGV (Robinson et al. 2011). Structural features were predicted using InterProScan v5.76-107.0 (Jones et al. 2014).

The identification of *Imagin* orthologs in isopods required careful examination because isopod *Imagin* sequences were substantially divergent from those of decapods. The Imagin ortholog from the deep-sea giant isopod *Bathynomus jamesi* (Yuan et al. 2022) was identified by TBLASTN search querying the PjImagin protein against the *B. jamesi* genome (Table 1; Table S4). Positive hits on scaffold JAJOZX010000594.1 exhibited the highest query coverage (63%) despite low amino acid identity (27.55%) and, as expected, were found nested between exons 14 and 15 of the predicted *MMUT* gene (Fig. 1b). The corresponding region (JAJOZX010000594.1:1094001-1104000) was extracted, and gene structures were predicted using Augustus v3.3.3 (https://bioinf.uni-greifswald.de/augustus/submission.php; Last accessed 2025 November) (Stanke et al. 2006) using the species model of *Bombus terrestris*, yielding a predicted *Imagin*-like gene (Supplementary Data S5). TBLASTN search querying the predicted protein against the *B. jamesi* genome assembly yielded a single strong hit, strongly suggesting that this locus is single copy (Table S5). The orthology of the *B. jamesi* Imagin-like gene with decapod *Imagin* was finally justified based on the phylogenetic analysis (Fig. 2). The predicted *B. jamesi* Imagin protein was further queried against isopod genome assemblies to identify Imagin orthologs in *Armadillidium vulgare* (Chebbi et al. 2019) and *Armadillidium nasatum* (Becking et al. 2019), which also lay between exons 14 and 15 of *MMUT* gene. The gene structure of *A. vulgare Imagin* was predicted using Augustus v3.3.3 (Supplementary Data S5), and the predicted *A. vulgare* Imagin protein sequence was mapped to the *A. nasatum* genome assembly using miniprot to locate the *A. nasatum Imagin* gene. *A. vulgare Imagin* was unambiguously a single-copy gene (Table S6), whereas TBLASTN search querying the predicted *A. nasatum* Imagin against the *A. nasatum* genome assembly yielded 2 other hits exhibiting 98% to 99% identity with 81% coverage (Table S7). To examine the possibility that this gene is an active Ginger1-like element, we compared the 3 relevant scaffolds (SEYY01021081.1, SEYY01003597.1, SEYY01017486.1) on the YASS web interface (https://bioinfo.univ-lille.fr/yass/; last accessed 2025 November) (Noé and Kucherov 2005). It turned out that the duplicated *Imagin* sequences were part of segmental duplications ranging from 7 to 11 kb, where duplicated sequences contained exons 10 to 14 of *MMUT* gene. No inverted repeats were observed in the neighborhood. These observations suggest the duplicated Imagin fragments in the *A. nasatum* genome assembly are not associated with the transposition activity of *Imagin*, if exist at all.

Additional isopod Imagin sequences were recovered from transcriptome shotgun assemblies (Jovović et al. 2024) (Table 1). The isopod Imagin proteins have a truncated C-terminal compared to those of decapods.

Imagin orthologs in amphipods could not be detected by TBLASTN search querying decapod or isopod Imagin protein sequences. However, the RefSeq annotation of the *Hyalella azteca* genome has a protein-coding gene (LOC108680262) between exons 14 and 15 of *MMUT* gene. Protein isoforms encoded by LOC108680262 have inconsistent names (“titin isoform X1” and “glutenin, low molecular weight subunit 1D1 isoform X2”), likely due to definitive functional domains and repetitive motifs toward the C-terminal. A TBLASTN search querying the protein sequence (XP_018024546.1) against the genome assembly returned no evidence of closely related homologs, further corroborating the view that this gene is single-copy (Table S8). XP_018024546.1 protein sequence was queried against genome and transcriptome shotgun assemblies of other amphipods, recovering additional 3 homologs (Table 1).

### Phylogenetic Analysis

The predicted amino acid sequences of malacostracan *Imagin* orthologs were aligned using MAFFT v7.525. The multiple sequence alignment was used for the maximum-likelihood phylogenetic analysis using IQ-TREE v2.3.6 (Minh et al. 2020). The resulting tree was visualized using FigTree v1.4.4.

A total of 97 representative DDE integrases and transposases were downloaded from the NCBI database (Table S9; Supplementary Data S6). The full-length protein sequences of Ginger1 elements were unavailable from (Bao et al. 2010). Therefore, we substituted Ginger1 elements with entries recovered as BLASTP hits showing >85% amino acid identity to the original sequences available in the Supplementary Data of Bao et al. (2010). The protein sequences were aligned by MAFFT v7.525 (Katoh et al. 2019), trimmed with trimAl v1.5.0 with “-automated1” option (Capella-Gutiérrez et al. 2009), and phylogenetic analysis was conducted with IQ-TREE v3.0.1 (Minh et al. 2020). The resulting tree was visualized using FigTree v1.4.4 (http://tree.bio.ed.ac.uk/software/figtree/).

### Structural Alignment

The Imagin protein structures were predicted using ColabFold v1.5.5 (Mirdita et al. 2022) (Supplementary Data S1). The multiple structural alignment of the Imagin proteins and representative integrases was generated by the “all against all structure comparison” workflow implemented in the DALI server (http://ekhidna2.biocenter.helsinki.fi/dali/; accessed November 2025) (Holm 2020).

### d*N*/d*S* Analysis

Selected Imagin CDS were retrieved or extracted from transcriptome or whole genome shotgun assemblies from the NCBI database (Table S10, Supplementary Data S4). The CDS were aligned using MAFFT v7.525, and the resulting alignment was used to build a maximum-likelihood phylogenetic tree using IQ-TREE v3.0.1. The alignment and the tree were input to HyPhy v2.5.78, using the FitMG94.bf batch file (https://github.com/veg/hyphy/issues/1573).

## Supplementary Material

evag010_Supplementary_Data

## Data Availability

The full-length cDNA sequence of *P. japonicus* Imagin (*PjImagin*) has been deposited in the DNA Data Bank of Japan (DDBJ) under the accession number LC877020.1. Supplementary Data S1 to S11 are available from FigShare (https://figshare.com/s/974df5db9ce49a29d5ad). Supplementary Data S1: AlphaFold2 protein structure models of Imagin proteins (Supplementary_Data_S1_AlphaFold_Models.zip); Supplementary Data S2: Raw Miniprot output files containing *Imagin* ortholog genomic alignments (Supplementary_Data_S2_Miniprot_Raw.zip); Supplementary Data S3: CDS alignments and HyPhy output files for dN/dS analysis (Supplementary_Data_S3_CDS_Alignments_dNdS.zip); Supplementary Data S4: Multiple sequence alignments and phylogenetic tree files for *Imagin* orthologs (Supplementary_Data_S4_Imagin_phylogeny.zip); Supplementary Data S5: Raw Augustus output files containing Imagin predictions of isopods (Supplementary_Data_S5_Augustus_Raw.zip); Supplementary Data S6: Multiple sequence alignments and phylogenetic tree files for DDE integrases and Ginger1 elements (Supplementary_Data_S6_DDE_phylogeny.zip); Supplementary Data S7: Bioinformatic scripts used in this study (Supplementary_Data_S7_Bioinformatic_methods.md).
