# Supplementary figures and images for: Imagin: An Integrase-Like Gene Conserved Across Malacostracan Crustaceans Derived From a Ginger1 DNA Transposon
Source: Genome Biol Evol. 2026 Jan 16;18(2):evag010. doi: 10.1093/gbe/evag010 (PMC12863088; doi:10.1093/gbe/evag010)

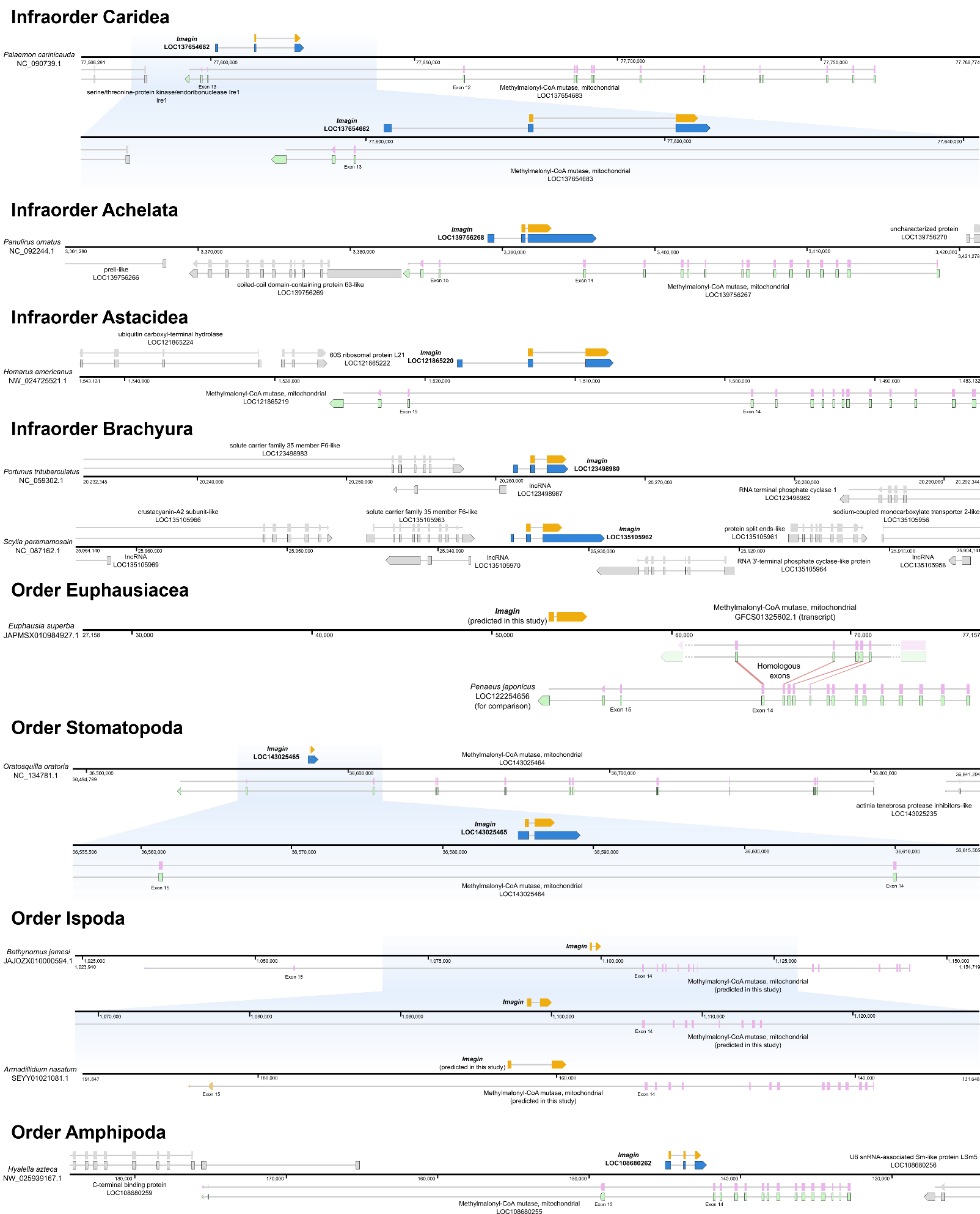


**Supplementary Figure 1. Genomic context of *Imagin* in malacostracans.**

Supplement: evag010_Supplementary_Data [file evag010_supplementary_data.zip › 2025-12-11_Supplementary_Figure.docx]
